# Job loss at home: children’s school performance during the Great Recession

**DOI:** 10.1007/s13209-020-00217-1

**Published:** 2020-05-29

**Authors:** Jenifer Ruiz-Valenzuela

**Affiliations:** Centre for Economic Performance, London School of Economics, London, UK

**Keywords:** Parental job loss, School performance, Great Recession, *I*20, *I*24, *J*63, *J*65

## Abstract

This paper studies the intergenerational impact of parental job loss on school performance during the Great Recession in Spain. Collecting data through parental surveys in a school in the province of Barcelona, I obtain information about the parental labour market status before and after the Great Recession. I can then link this information to repeated information on their children’s school performance, for a sample of over 300 students. Using individual fixed effects, the estimates show a negative and significant decrease on average grades of around 15% of a standard deviation after father’s job loss. These results are mainly driven by those students whose fathers suffer long unemployment spells. In contrast, the average impact of mother’s job loss on school performance is close to zero and non-significant. The decline in school performance during the Great Recession after father’s job loss, particularly among disadvantaged students, could result in detrimental long-term effects that might contribute to increased inequality. This could be an important and underemphasised cost of recessions.

## Introduction

One of the most distinct features of the past Great Recession was the high incidence of job losses on either side of the Atlantic. In Europe (EU-27), there were almost 7 million fewer people in paid employment in 2012 than in 2008.^1^ In the USA, the Displaced Workers Survey data show a record high rate of job loss in the 2007–2009 period, with the rate of unemployment peaking at 10% in the first quarter of 2010 (Farber 2015). In addition to the high level of job destruction, the Great Recession was also characterised by low reemployment rates. The available evidence indicates that job losers suffer short-run earning losses that persist in the long run, have prevalent feelings of job insecurity, worse physical and mental health, an increased risk of divorce and, upon re-employment, a moderate increase in workplace injuries.^2^ Other than the negative consequences borne by the worker, this dramatic weakening of the labour market has thus the potential to generate serious spillover effects for other members of the household, particularly for children. This paper analyses the intergenerational impact of parental job loss by investigating how job losses that occurred during the Great Recession in Spain affect children’s school performance. Given the high unemployment incidence during the Great Recession, the effect of parental job loss on children’s educational outcomes during this period is potentially large. This could be an important and underemphasised cost of recessions.

Most of the negative consequences of job loss have a direct effect on variables that are normally seen as inputs of the production function of cognitive achievement. These include impacts on parental income, shifts in parental time investments and deteriorated mental and physical parent’s health. For instance, reduced household income following parental job loss could alter the financial resources available to children’s education. Parental job loss could also be linked to changes in both the quantity and quality of time devoted to children, as well as a shift in the time allocation devoted to childcare in two-parent households. Moreover, it could be challenging to shield children from the harmful consequences on parent’s mental health that could be felt after parental job loss. Importantly, the context of these job losses might exacerbate the potential negative consequences for children’s cognitive development. As Kalil (2013) noted in her review of the effects of the Great Recession on child development, we know very little about the Great Recession’s impacts on children. The results in this paper will shed light on how a deep economic recession disproportionately affecting some sectors in the economy impacts the educational outcomes of students whose fathers are severely hit by the downturn.

As Rege et al. (2011) note, estimating a causal relationship between parental job loss and children’s outcomes is subject to two main challenges: finding a source of exogenous variation for parental job loss and the scarcity of appropriate data. Data sets like the Panel Study of Income Dynamics (PSID) or the Survey of Income and Program Participation (SIPP), do not offer precise and repeated information on student outcomes; i.e. most of the times, the only school-related outcome available is the maximum education level reached at a certain age. Another important contribution of this paper is to exploit a panel data set put together by the author, with detailed information for over 300 students in a school in the province of Barcelona.^3^ Collecting data through parental surveys, I obtain information about the parental labour market status and, for those losing the job during the Great Recession, the date and reason of job loss. I can link this information to repeated information on their children’s school performance for academic years 2008–2012. This is crucial, since it allows for a children fixed effect methodology.

As will be made clear in Sect. 2, key contributions to the literature have used plant closures to identify the causal impact of father’s job loss on their children’s outcomes. However, Card et al. (2013) show that there is a non-random selection of workers into closing or struggling firms. One of the ways in which this paper addresses this challenge is by controlling for all those unobserved, time-invariant parental characteristics that might be behind the selection of workers into losing their jobs. Importantly, the results using the fixed effect model are compared to those obtained in a fashion similar to that used in the plant closure literature, and those coming from value-added regressions. The findings show that the estimates coming from the two latter strategies are considerable larger in magnitude than those obtained using fixed effects. This suggests that if the data are not sufficiently rich to control for potential determinants of school performance (which are in turn linked to parental job loss), previously used strategies could render estimates that suffer from potential bias due to selection of whom is laid off.

**Fig. 1:**
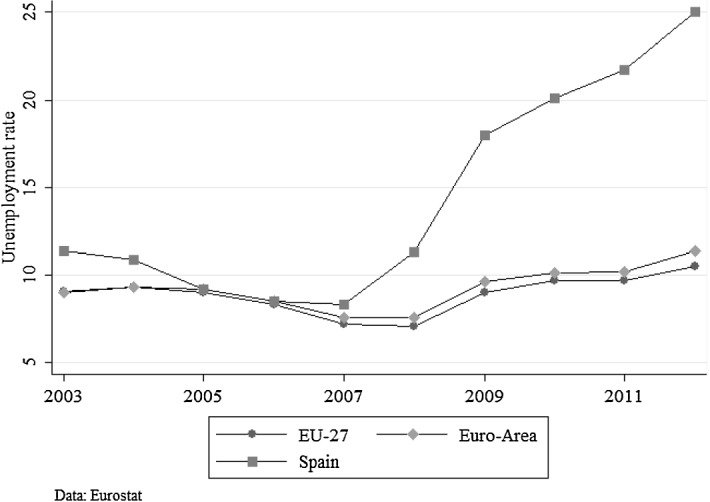
Unemployment rates *Note*: Unemployment rates in percent, for the EU-27, euro area and Spain; from 2003 to 2012 *Source*: Eurostat

The proportion of job losses occurring in the sample during the period is very similar to the pattern seen in the Spanish labour market. Figure 1 shows the unemployment rates in the EU-27, euro area and Spain. The figure shows how the Spanish unemployment rate reached its lowest point in 2007 and started increasing dramatically thereafter, approaching 25% in 2012. I use this fact to try to get a closer approximation of causal effects. In this sense, I define the treatment group by using an event that could potentially resemble a natural experiment (i.e. students whose parents lost their jobs due to the Great Recession). However, even during a recession, those who lose their jobs—or lose their jobs first—might have different characteristics than those who keep them (or keep them for longer). The use of student fixed effects (and additional checks that suggest that there is indeed exogenous timing of job loss in the sample used in this paper) helps overcome potential selection into job loss.

I find that the impact of father’s job loss on the average grade is negative and statistically significant. Paternal job loss entails an average decrease in children’s grades of around 15% of a standard deviation. The average impact of mother’s job loss on school performance is close to zero and non-significant. These results are in line with those reported by Rege et al. (2011) that argue that a disparate effect of job loss across fathers and mothers is consistent with recent empirical studies documenting that the mental distress experienced by displaced workers is generally more severe for men than for women [see, for instance, Kuhn et al. (2009)]. Additionally, the results suggest that the negative impact of father’s job loss on school performance is mainly driven by those fathers that suffer longer unemployment spells. Related to this, the effect of father’s job loss appears to be largely concentrated among children of already disadvantaged families in terms of father’s education.

One of the potential mechanisms that could be driving the results is the observed decline in income after father’s job loss. However, the heterogeneous results for different subgroups are not fully explained by different income losses. Moreover, it is important to note that these results are obtained for students that are enrolled in the same school during the period of observation. The observed reduction in income cannot be linked, therefore, to changes in the school attended after job loss. An alternative channel by which reductions in income could partly explain these results is if income declines after father’s job loss entail higher stress or financial anxiety and uncertainty for affected individuals and households; an effect reported in some social psychology and health economics research [see, for instance, Lim and Sng (2006) and Kuhn et al. (2009)].^4^

I explore the robustness of these results in a number of ways. Placebo tests show that the average grade prior to father’s job loss is not affected by future job losses experienced by the father. Additionally, the negative effect of father’s job loss does not seem to be driven by those students whose fathers had shorter tenure at the firm prior to job loss, but rather, by those fathers that had a more stable situation prior to losing their jobs. Also, the average grade does not exhibit a negative trend prior to treatment and the results are robust to the inclusion of group-and-year, as well as group-and-stage of education specific effects.

The structure of the paper is as follows. Section 2 reviews the literature most closely related to this paper. Section 3 describes the original data set used in the paper while Sect. 4 presents the empirical strategy. Section 5 shows the main results and robustness checks, and Sect. 6 concludes.

## Previous literature

Family background characteristics are considered important inputs determining child development.^5^ One of the main features of family background is the labour market status of parents and, in particular, experiences of transitions in and out of employment. As a result, an emerging literature has tried to identify whether parental job loss has an effect on the education attainment and school performance of children. I summarise most of the existing papers in the economic literature in Table 1. The articles are divided by their use of the data available.

**Table 1:** Summary of studies of parental job loss and the educational outcomes of their offspring (in the economic literature)

Paper	Source and years	Country	Age children	Treatment	Outcome variable	Estimates are...	Results
*Use of the data: cross-sectional (outcome variable observed once for a given individual)*							
Kalil and Wightman (2011)	PSID, 1968:2005. Those born and turning 21 within panel	USA	21	Head ever reported involuntary JL by time child is 21	Any postsecondary educ by age 21	OLS and probit estimates	Parental JL $\downarrow$ prob of postsecondary education by 10pp
Gregg et al. (2012)	BCS: panel of indiv born April 1970	UK	16	Fathers in industries with 20% employment loss in 80s recession	GCSE attainment	OLS estimates with a measure of prior attainment	$\downarrow$ 18 grade points less, or half a GCSE at grades $\hbox {A}^{*}$-C
Pan and Ost (2014	PSID: indiv born between 1970 and 1985	USA	18–20	Parental layoff at ages 15–17 versus 21–23 (control)	Higher education (HE) enrollment	Linear probab estimates	Parental JL $\downarrow$ prob of HE enrollment (10 pp)
*Use of the data: repeated cross sections (outcome variable observed once for a given individual. Authors pool different cross sections)*							
Rege et al. (2011)	Admin data, 2003:2007	Norway	16 (year 10)	JL (in school years 8–10) from PC	Summary measure of 10 subjects	Pooled OLS	FJL $\downarrow$ average grade 6.3% SD. No effect MJL
Coelli (2011)	SLID: 4 panels (1993–2007)	Canada	16–20	JL (ages 16–18); perm layoff or firm closure	College enrollment	Linear probab estimates	JL main income earner $\downarrow$ prob univ enrollment (10pp)
Hilger (2016)	Admin tax records: from 2000 to 2009	USA	18–22	Father’s layoffs (taking up UI) before college	College enrollment	DD: uses time of JL in control and survivor sample	Father’s layoff $\downarrow$ annual college enrollment by 1%
*Use of the data: panel data (outcome variable observed more than once for a given individual)*							
Kalil and Ziol-Guest (2008)	SIPP: Panel of 1996	USA	5–17	Involuntary JL 24 months prior to measure outcome)	Grade retention	Logit estimates with lagged dependent vble	FJL doubles odds of grade retention. No effect MJL
Stevens and Schaller (2011)	SIPP: panels of 1996–2001–2004	USA	5–19	Involuntary JL of HH (after wave 1)	Grade retention (and expulsion)	FE estimates	Grade retention $\uparrow$ 15%
This paper	Own data collection: 2008:2012	Spain	3–16	Involuntary JL during Great Recession	Summary measure: average grade	FE estimates	FJL $\downarrow$ average grade by 15% SD No effect MJL

JL, Job Losses; FJL(MJL), Father(Mother)’s job losses; BCS, British Cohort Study; GCSE, General Certif of Secondary Education; SIPP, Survey of Income and Program Participation, SLID, Survey of Labour and Income Dynamics; PSID, Panel Study of Income Dynamics; PC, plant closure; UI, unemployment insurance benefits; HH, household head; DD, Diff-in-Diff; FE, fixed effects; pp, percentage points; SD, standard deviation; HE, higher education

The results of the articles in the first panel of Table 1 are obtained by using the data in a cross-sectional fashion. Kalil and Wightman (2011) and Pan and Ost (2014) study the impact of parental layoff on any postsecondary education by age 21 and higher education enrolment, respectively, in the USA. The main problem with the identification strategy in Kalil and Wightman (2011) is that those suffering involuntary job losses are systematically different from those that remain continually employed. Pan and Ost (2014) try to overcome this problem by using variation in the timing of parental lay-off. In their study, all families experience a lay-off at some point. The last study in this group by Gregg et al. (2012) uses the British Cohort Study to construct their group of displaced fathers. They combine information on whether fathers worked in industries hit hard by the 1980’s crisis with whether they were either out of work or employed in a different industry by 1986. They argue that the extent to which the industry is hit (and hence the likelihood that the father is displaced) is deemed exogenous to the father’s unobserved characteristics and to the child’s educational development. However, there is still the possibility that workers with different unobserved characteristics sort themselves into different industries.

In the second panel of Table 1, I summarise studies that use repeated cross sections (i.e. the outcome variable is still observed once for a given individual). In this group, both Coelli (2011) and Hilger (2016) study college enrolment. Coelli (2011) constructs the treatment group as those students whose parents (the main income earner) suffered a job loss due to permanent lay-off or firm closure. It is common in this literature to assume that this type of parental job loss (as opposed to being fired, for instance) is exogenous to the worker. The same strategy is used by Rege et al. (2011) to study the impact of father’s job loss on an average measure of their children’s school performance at age 16. Hilger (2016)’s findings, however, show that firm closures generate much larger effects on child outcomes, but that these larger effects stem entirely from selection on unobservables into employment at closing firms.

In the last panel of Table 1, the studies—including the present one—use panel data and can therefore observe the outcome variable in more than one period for a given individual. If the characteristics that drive assortativeness into different firms are constant over time (i.e. level of education, how productive an individual is, permanent character traits, etc.), then the use of panel data has an advantage when trying to identify the causal effect of parental job loss (although only Stevens and Schaller (2011) and this study take advantage of the panel dimension in the data to obtain fixed effect estimates). Moreover, the outcome variable in both Kalil and Ziol-Guest (2008) and Stevens and Schaller (2011) is grade retention (a variable that, even if important to measure attainment, is a low-frequency one).

In summary, the only prior study that produced fixed effect estimates (Stevens and Schaller 2011) used a low-frequency outcome measure and did not have the additional push into job loss given by the Great Recession. I combine different positive aspects of the papers just reviewed to try to get a closer approximation of the causal impact of father’s job loss on school performance during a recession.

## Data

### Data collection and validity

The data requirements needed to assess the causal impact of parental job loss on the educational attainment of their offspring are very demanding. There are very few panel data sets that allow linking repeated school performance measures with repeated measures of parental labour market status. In the case of Southern Europe, where the impact of the Great Recession in terms of job loss has been particularly high (and therefore the question becomes even more relevant), panel data sets of this nature do not exist. This is why the empirical analysis uses an original data set collected to address the research questions that motivate this article. Excluding students in post-compulsory education, the data set contains information on the parental labour market situation (collected through a survey) and school performance of 358 students between the ages of 3 and 16 in a school in the province of Barcelona.

**Fig. 2:**
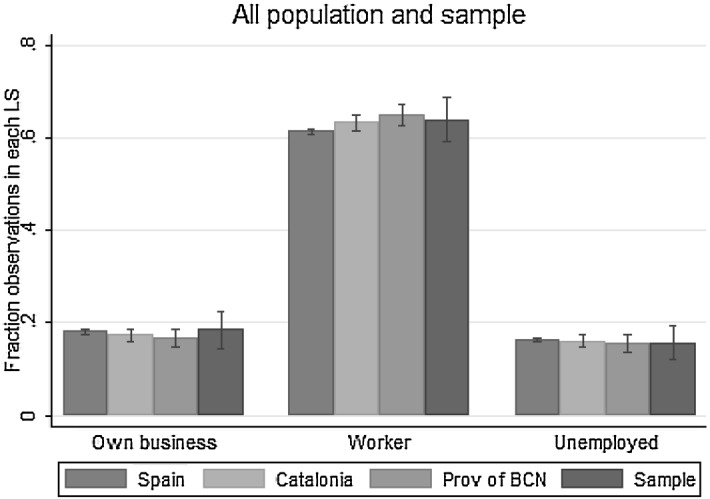
Father’s labour market status. *Note*: Fraction of the population in each geographic area (Spain, Catalonia, Province of Barcelona) by Labour Status (LS), with 95% confidence intervals. The population data refer to the first quarter of 2012 of the Spanish Labour Force Survey (LFS)

Concerns about the external validity of the results will arise when these are produced by findings from one school. However, previous studies based on data from single institutions have generated important insights in leading outlets [see, for instance, Angrist et al. (2010), Dobbie and Fryer (2015) and Bursztyn et al. (2017)]. For the purpose of this paper, it is crucial to note that the school examined is an average school in the region of Catalonia, in terms of both school performance (measured by performance in standardised tests) and the labour market status of parents.^6^ Importantly, the labour market status of fathers at the point of data collection is remarkably similar to the labour market status of fathers (with children aged 0–20) observed in Spain, Catalonia and the province of Barcelona. Using data from the first quarter of the 2012 Spanish Labour Force Survey (LFS), I present the distribution of father’s labour market status in Fig. 2. The labour market status is divided into three categories: those who own a business, those work for a firm and those who are unemployed. The sample distribution is almost identical to the distribution in Spain, Catalonia and the province of Barcelona. The results of Pearson Chi-square tests show that there are no significant differences in the frequency distribution of father’s labour market status between the sample and these populations.^7^

About 50% of the parents in the school participated in the survey. While this could seem problematic, there are no remarkable differences between the sample and the overall school population in terms of the age of the cohorts responding, the student’s month of birth or their home postcode. The available evidence suggests that there are no remarkable differences in terms of school performance either, once students with an immigrant background are excluded from the sample (see Appendix A, Sect. A.1.3).

### Data collection and recall bias

On the parental side, I designed a survey to collect information on their personal characteristics (age, level of education, civil status, etc.) and labour market-related data (labour market status, characteristics of the job, reasons for job loss or for switching jobs, etc.). For logistic reasons and in order to increase the response rate, I asked both parents to answer the survey as long as they were living in the same household as the children. The information on parental labour market status (and job characteristics if employed) was collected retrospectively. That is, in February 2012 (when the survey was distributed), I asked parents about their labour market situation and job characteristics on the first of January 2012, January 2010 and January 2008. If their employment situation changed at some point after January 2008, parents were asked to provide information on the month and year when this change occurred. With information about these three points in time and the dates regarding employment status changes, the labour market situation for parents is obtained for the five periods in which their offspring grades are observed.^8^ I offer a more detailed description of the data collection, survey and item non-response in Appendix A (Sect. A2).

Job loss data collected retrospectively face potential issues related to recall bias (see, for instance, the findings by Evans and Leighton (1995) when analysing recall bias issues in the context of the Displaced Worker Survey of the US Bureau of Labour Statistics). According to Evans and Leighton (1995), the bias results from the fact that respondents’ memory of displacement erode over time. In order to minimise the incidence of recall bias, I collected information about the labour market status of parents on the first of January 2012, January 2010 and January 2008. Fixing the date at the beginning of the year should help parents to remember their employment situation at that particular point in time. In order to understand whether recall bias might be a potential problem in the sample collected for the purposes of this paper, I compare the unemployment rates in the sample in January 2008 and January 2012, with those in the province of Barcelona in the first quarter of 2008 and 2012, using the Spanish LFS data. That is, I compute the fraction of males in the province of Barcelona (with children aged 0 to 20) that were unemployed in the first quarter of 2008 and 2012. At the beginning of 2008, 4% of those males were unemployed in the province of Barcelona, compared to almost 3% in the sample. By the beginning of 2012, the unemployment rate for fathers in the sample had increased to almost 16%, whereas the unemployment rate for fathers in the province of Barcelona was about 15%. The Spanish Labour Force data are not collected retrospectively, i.e. the respondent is asked about his/her employment situation in the week prior to the survey. Therefore, recall bias concerns are not an issue in the LFS. The fact that the unemployment rates in the sample and in the province of Barcelona are very similar, both at the beginning and at the end of the period, suggests that problems of recall bias are likely to be unimportant in the sample collected.

### Treatment group definition, outcomes and sample restrictions

**Treatment group definition** I explore three different treatment definitions. First, a strict definition of job loss is used, where job losers are defined as those fathers involuntary losing their jobs during the crisis. Second, given the sample size and in order to increase the number of treated clusters, I use a more loose definition of job loss: Job losers are defined as those fathers involuntarily losing their jobs during the crisis, but also those closing their own business or unemployed fathers who state that the reason behind their unemployment (after having been employed during the period) is voluntary. Finally, the third definition only considers job losses coming from firm downsizing or closures during the period of observation and excludes from the working sample those children whose fathers suffer other types of job losses. Using these definitions, I first analyse the impact of father’s job loss and address later the role of mother’s job loss in a similar fashion.

**Outcome definition** The school granted access to the grades obtained by those students whose parents answered the questionnaire (from academic year 2008 to academic year 2012). The format of the grades for each stage of education is described in Sect. A3 in Appendix A. After some transformations to homogenise grades between stages of education, all the students in the sample have grades ranging from 1 to 5 (where 1 means that the student has failed the subject and 5 is the best possible grade). Based on this information, I construct a summary measure of each student’s performance during the academic year. In particular, the main measure used throughout the analysis is the average grade obtained each academic year by each student. This measure is obtained by averaging the student’s grades in all subjects taken during the three terms in a given academic year.

**Sample restrictions** As in Rege et al. (2011), the results are limited to the sample of students whose parents have stayed together during the period under analysis (332 students).^9^ Additionally, in order to be able to compare the school performance of students before the start of the Great Recession and at the end of the period analysed, I need to work with the sample of students that were enrolled in the school since the academic year 2008. This reduces the available sample to 193 students, but I also report results for the full sample of 332 students observed in 2012.^10^

I apply two further exclusion criteria to create the final sample. First, given that the sample does not seem to be representative of students with an immigrant background (see Sect. A.1.2 in Appendix A), I exclude students whose fathers do not hold Spanish citizenship (10 students). The second exclusion criterion is important for the identification strategy and internal validity and has to do with the employment status of fathers in the first period of observation. Following Stevens and Schaller (2011) the sample only includes students whose fathers were employed in January 2008. That is, all the students in the working sample have their fathers employed in the first period under analysis. After applying this restriction, the main analytic sample consists of 178 students in compulsory education whose grades were observed for all five academic years from 2008 to 2012 and whose fathers were employed at the beginning of the crisis (in academic year 2008). Furthermore, the fathers in this sample are all Spanish citizens who were present in the family home throughout the period.

### Descriptive statistics

**Fig. 3:**
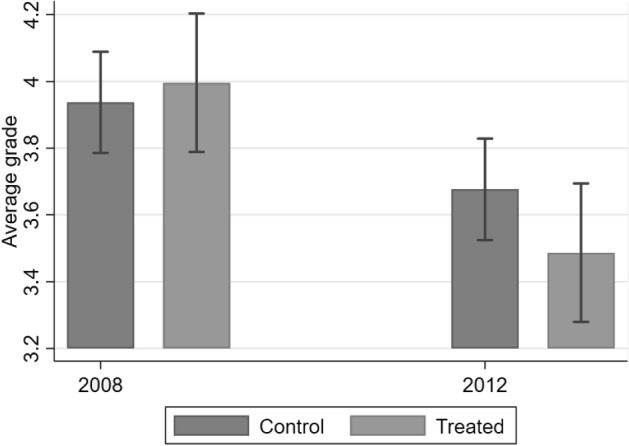
Average grade pre- and post-treatment. *Note*: Mean (given by the height of the bar) and 95% confidence intervals. The average grade in a given academic year goes from 1 to 5 (where 1 means that the student has failed the subject and 5 is the best possible grade). The sample used is the one after applying the restrictions explained in the main text. Looser definition of job loss used here to define treated students, to give an overview of all students whose fathers lose their jobs during the period under analysis. Results are very similar using the strict definition of job loss

Figure 3 shows the average grade for treated and control students in the academic years 2008 and 2012.^11^ In the academic year 2008, when all fathers in the sample were employed, there were no significant differences in the average grade between treated and control students. By 2012, both treated and control students have suffered a decrease in school performance as they progress in the education system (this is captured by year and stage of education dummies in the regression analysis described below). However, the group of students that have been affected by paternal job loss suffers a larger decrease. Without additional controls, the difference in means is not statistically significant at conventional levels, but will become so when standard errors are reduced by further controlling for year, stage of education dummies and student fixed effects in the regression analysis.^12^

**Table 2:** Descriptive statistics. Children and household characteristics in 2008

	C.1	C.2	C.3	C.4
	Control (C)	Treated (T)	All	Difference (T-C)
*Outcome measure*				
Average grade	3.937	3.996	3.955	.058
	(0.823)	(0.751)	(0.801)	(.132)
*Children characteristics*				
Born Q1	0.242	0.185	0.225	$-$ 0.057
	(0.430)	(0.392)	(0.419)	(0.068)
Born Q2	0.298	0.241	0.281	$-$ 0.058
	(0.459)	(0.432)	(0.451)	(0.071)
Born Q3	0.274	0.407	0.315	0.133*
	(0.448)	(0.496)	(0.466)	(0.079)
Female	0.524	0.593	0.545	0.068
	(0.501)	(0.496)	(0.499)	(0.083)
Age	8.306	8	8.213	$-$ 0.306
	(2.697)	(2.503)	(2.636)	(0.405)
First child	0.548	0.481	0.528	$-$ 0.067
	(0.500)	(0.504)	(0.501)	(0.061)
*Household characteristics*				
Number of children	1.935	2.093	1.983	0.157
	(0.506)	(0.759)	(0.596)	(0.147)
Household size	3.927	4.056	3.966	0.128
	(0.528)	(0.787)	(0.619)	(0.154)
Stable civil status	0.960	0.926	0.949	$-$ 0.034
	(0.198)	(0.264)	(0.220)	(0.060)
Mother has a job	0.798	0.852	0.815	0.053
	(0.403)	(0.359)	(0.390)	(0.075)
Family lives close to school	0.589	0.519	0.567	$-$ 0.070
	(0.494)	(0.504)	(0.497)	(0.101)
Language spoken at home:	0.589	0.537	0.573	$-$ 0.052
Spanish	(0.494)	(0.503)	(0.496)	(0.101)
House: owned	0.395	0.389	0.393	$-$ 0.006
	(0.491)	(0.492)	(0.490)	(0.097)
House: paying mortgage	0.573	0.481	0.545	$-$ 0.091
	(0.497)	(0.504)	(0.499)	(0.101)
House: rented	0.0161	0.0926	0.0393	0.076
	(0.126)	(0.293)	(0.195)	(0.064)
*N*	124	54	178	

First (second) line for each variable corresponds to its mean (standard deviation). *, **, *** denote significance at the 10%, 5% and 1% levels. The 4th column shows the difference in means for treated and control individuals and clustered standard errors (at the family level) for this difference (in parentheses). There are 137 family clusters (40 correspond to treated families). Values are for academic year 2008, except for the *household size* and *house dummies* variables (information for these variables was not collected for 2008, so values correspond to 2012). Looser definition of job loss used to define treated students, to give an overview of all students whose fathers lose their jobs during the period under analysis. Results are very similar using the strict definition of job loss

**Table 3:** Descriptive statistics. Father characteristics in 2008

	C.1	C.2	C.3	C.4
	Control (C)	Treated (T)	All	Difference (T-C)
Education	0.419	0.315	0.388	$-$ 0.105
Beyond high school	(0.495)	(0.469)	(0.489)	(0.097)
Age	40.80	41.96	41.15	1.165
	(4.788)	(4.526)	(4.728)	(0.840)
High income	0.765	0.563	0.706	$-$ 0.203**
	(0.426)	(0.501)	(0.457)	(0.100)
	115	48	163	
Income missing	0.0726	0.111	0.0843	0.039
	(0.260)	(0.317)	(0.279)	(0.061)
*Labour market characteristics*				
Own business	0.242	0.315	0.264	0.073
	(0.430)	(0.469)	(0.442)	(0.093)
Industry	0.250	0.413	0.296	0.163
	(0.435)	(0.498)	(0.458)	(0.102)
	116	46	162	
Construction	0.155	0.370	0.216	0.214**
	(0.364)	(0.488)	(0.413)	(0.098)
	116	46	162	
Tenure since year	1994.4	1998.6	1995.7	4.213***
	(6.875)	(6.769)	(7.095)	(1.276)
Permanent contract	0.989	0.714	0.915	$-$ 0.275***
	(0.103)	(0.458)	(0.280)	(0.101)
	94	35	129	
Full time work	0.974	0.911	0.957	$-$ 0.063
	(0.159)	(0.288)	(0.204)	(0.054)
	117	45	162	
High motivation	0.784	0.696	0.758	$-$ 0.088
	(0.414)	(0.465)	(0.430)	(0.098)
	111	46	157	

First (second) line for each variable corresponds to its mean (standard deviation -SD-). *, **, *** denote significance at the 10%, 5% and 1% levels. The 4th column shows the difference in means for treated and control individuals, and in parentheses, the clustered standard error for the difference (clustered at the family level). There are 137 family clusters, of which 40 clusters correspond to treated families. A third row with the number of observations is shown when a variable has missing values. Values are for academic year 2008. High motivation equals 1 if in 2008 the father had a level of motivation at work of 4 or 5 (measured in a scale of 1–5, where 5 means very motivated). Looser definition of job loss used here to define treated fathers, to give an overview of all students whose fathers lose their jobs during the period under analysis. Results are very similar using the strict definition of job loss

I show descriptive statistics for the working sample of 178 students, measured in 2008 (unless otherwise stated) in Tables 2 and 3. In each table, the first three columns report means and standard deviations for different variables for the control, treated and overall analytic sample, respectively. In Column 4, I report the difference in the mean for control and treated individuals in the first row, and clustered standard errors for the difference (clustered at the family level) in the second row.

Table 2 contains descriptive statistics for several child and household characteristics. I first show that the average grade for treated and control students was very similar in 2008. Additionally, except for the quarter of birth dummies, there are no statistically significant differences between treated and control students in terms of gender, age or whether they are the first-born child. In terms of household characteristics, there are no significant differences between treated and control students in the average number of children living in the home, household size (measured in 2012), the language spoken at home, whether the mother was living with the partner or was married (i.e. stable civil status), whether the mother had a job, whether the family lived in a neighbourhood close to the school area or the house property status.^13^

As pointed out in Sect. 1, it is unlikely that people losing their jobs during the Great Recession in Spain are randomly selected across the whole employed population. This is reflected in Table 3, which shows descriptive statistics for fathers.^14^ Fathers of treated students already had a lower level of income in 2008, and a higher fraction was working in the industry and construction sectors.^15^ Moreover, treated fathers had fewer years of tenure at the firm and a lower share of permanent contracts. There are no significant differences in the level of education of the fathers of treated and control students. It is also interesting to note that there were no significant differences in their level of motivation at work in 2008.^16^

All in all, the information in Table 3 seems to suggest that, without controlling for student (worker) fixed effects, job loss during the Great Recession in Spain cannot be considered to be as good as randomly assigned.

## Empirical strategy

Let $Y_{it}$ equal the standardised average grade for child *i* in academic year *t*.^17^ Let $D_{it}$ denote a dummy variable that equals 1 from the year the father involuntarily loses his job. On account of the sample restrictions outlined in Sect. 3, this indicator equals 0 in the academic year 2008 for all the students, since all the fathers in the analytic sample are employed at the beginning of the Great Recession. For control students, this dummy will take a value of 0 in every period. For treated students, it will be 1 from the year the father loses the job (i.e. the treatment is an absorbing state).

Using the panel nature of the data, and the notation in Angrist and Pischke (2008), the following fixed effects model can be estimated:

1 $$\begin{aligned} Y_{it}= & {} \alpha _{i} + \lambda _{t} + \rho D_{it} + X'_{it}\beta + \epsilon _{it} \end{aligned}$$

2 $$\begin{aligned} \alpha _{i}= & {} \alpha + A'_{i}\gamma + X'_{i}\phi \end{aligned}$$

where $\alpha _{i}$ is the individual fixed effect, $\lambda _{t}$ represents a vector of year dummies, $X_{it}$ is a vector of observed time varying covariates not affected by the job loss itself (i.e stage of education dummies) and $\epsilon _{it}$ is the error term. Equation 2 shows static determinants of the student’s average grade that will be captured by the inclusion of individual fixed effects: $\alpha$ is the constant of the model, $X_{i}$ is a vector of observed time invariant covariates affecting the educational outcomes of the child, both at the child, household and father/mother level (like the level of education of the father, permanent wealth of the household, etc.), and $A_{i}$ is a vector of unobserved but fixed confounders capturing, among other things, the unobserved ability of the student. As Angrist and Pischke (2008) point out, the key to fixed effects estimation is the assumption that the unobserved $A_{i}$ appears without a time subscript.

The parameter of interest, $\rho$, captures the full effect of father’s job loss on the average grades obtained by their offspring. This may incorporate changes in income, civil status, etc., that happen as a result of parental job loss. Their role as potential mechanisms driving the results will be discussed in Sect. 5. In this paper, there is no attempt to incorporate all the determinants of cognitive achievement in the model, as Todd and Wolpin (2003) would put it. Instead, I make use of an event (the Great Recession) that arguably provides a source of exogenous variation for father’s job loss once student fixed effects are accounted for (or what is the same given the nature of the sample, once worker’s fixed effects are accounted for).

In models with panel data and fixed effects, the data are only informative about the impact of binary regressors on individuals for whom the value of the regressor changes over the period of observation. As Imbens and Angrist (1994) point out, the local average treatment effect (LATE) is analogous to a regression coefficient estimated in linear models with individual fixed effects using panel data. Therefore, rather than identifying average population effects, the estimates in this paper could be seen as measuring a local average treatment effect, namely the effect of father’s job loss for those students whose fathers lost their jobs due to the Great Recession.

**Fig. 4:**
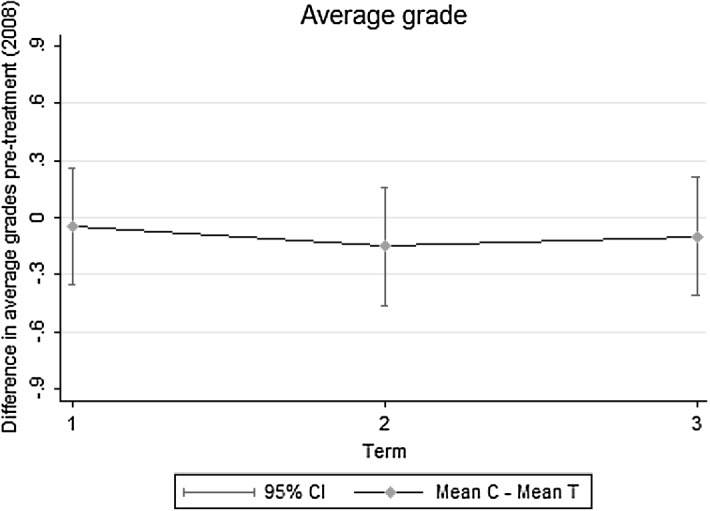
Difference in the average grade between control and treated students in 2008. *Note*: Difference in the average grade (control students (C) − treated students (T)) in the three terms of the academic year 2008 (i.e. prior to job loss for any treated students in the sample). The sample used is the one after applying the restrictions explained in the main text. Looser definition of job loss used here to define treated students, to give an overview of all students whose fathers lose their jobs during the period under analysis. Results are very similar using the strict definition of job loss

As mentioned above, the treatment is defined as an absorbing state. This is because conditioning on student fixed effects, father’s job loss in the sample is more likely to be unrelated to unobserved worker’s characteristics than finding a job afterwards.^18^ The main assumption in the paper is that conditioning on worker fixed effects the Great Recession generates employment shocks that are random in their timing. This assumption hides one potential risk for the consistency of the estimates, since it cannot be ruled out that unobserved time variant variables might simultaneously be affecting the probability of father’s job loss and the grades of their offspring. A major concern for the estimation strategy is given by the fact that fathers who lost their jobs during the period under analysis could have been on a negative trajectory in the labour market prior to 2008. And this, in turn, could have had an impact on average grades even before the beginning of the Great Recession.^19^ I use the data on grades in each term in 2008 to show that prior to treatment for any of the students in the analytic sample, there is no evidence of a differential trend in the average grade (see Fig. 4). Another way to address this issue is to check whether the impact of father’s job loss is mainly driven by students whose fathers had a lower labour market attachment prior to losing their jobs. I show in Sect. 5 that this does not seem to be the case. Additionally, the main characteristics of workers who lost their jobs in the first period of the crisis (2009–2010) and those that lost their jobs in the second period (2011–2012) are not significantly different from each other. That is, it seems that the Great Recession was not affecting different types of workers throughout the period (see Table 4).

**Table 4:** Characteristics of treated fathers in the first and second period after job loss. All job losers

	C.1	C.2	C.3	C.4
	2009–2010	2011–2012	All FJL	Difference
Father’s educ	0.304	0.270	0.292	$-$ 0.034
Beyond high school	(0.464)	(0.450)	(0.457)	(0.120)
Father’s age	40.80	41.43	41.02	0.635
	(5.155)	(6.589)	(5.674)	(1.451)
Father works in a firm in 2008	0.768	0.676	0.736	$-$ 0.092
	(0.425)	(0.475)	(0.443)	(0.114)
Sector: industry	0.359	0.306	0.340	$-$ 0.054
	(0.484)	(0.467)	(0.476)	(0.126)
Sector: construction	0.359	0.389	0.370	0.030
	(0.484)	(0.494)	(0.485)	(0.126)
Sector: services	0.281	0.306	0.290	0.024
	(0.453)	(0.467)	(0.456)	(0.119)
*N*	69	37	106	

First (second) line for each variable corresponds to its mean (standard deviation -SD-). *, **, *** denote significance at the 10%, 5% and 1% levels. The 4th column shows the difference in means for treated and control individuals, and in parentheses, the clustered standard error for the difference (clustered at the family level: 74 family clusters (71 for variables with missing values)). There are 6 missing values in the sector of activity variables (64 individuals in period 2009–2010, and 36 in 2011–2012). Values are for the academic year 2008. Looser definition of job loss used here to define treated fathers, to give an overview of all students whose fathers lose their jobs during the period under analysis. Results are very similar using the strict definition of job loss

Even if treated students do not seem to be on a different trend prior to father’s job loss, a further potential concern with the specification in Eq. 1 is that estimates might be driven by negative trends in school performance for particular groups of students.^20^ These negative trends could be due to differential effects by subgroups as the crisis unfolded, or due to differential trends in parental investment as the child advances within the education system. In order to take these concerns into account, the model is augmented by (1) interacting the year dummies with certain group specific characteristics; (2) interacting the stage of education dummies with certain group specific characteristics, respectively. This is represented by $\delta _{tj}$ in Eq. 3:

3 $$\begin{aligned} Y_{ijt}= \alpha _{i} + \lambda _{t} + \delta _{tj} + \rho D_{it} + X'_{it}\beta + \epsilon _{ijt} \end{aligned}$$

where *j* stands for different group characteristics measured in 2008, like father’s education, father’s income category, father’s sector of work or the student’s gender.^21^

Estimates of $\rho$ could also be biased for reasons related to attrition. For instance, estimates would be biased if students affected by father’s job loss had changed or left school by 2012. Given that we could only distribute the survey to those students enrolled in the school where the data were collected in 2012, it could be that prior to 2012 students affected by parental job loss had dropped out from this school and enrolled in a public one. This does not seem to be a cause for concern since the dropout rate for the school in both kindergarten and primary school grades are quite stable during the period of observation and less than 1% per year. In compulsory secondary school, the average annual dropout rate is a bit larger at around 3%. However, rather than increasing, the dropout rate decreases from academic year 2008 to academic year 2011 (i.e. the last year for which data on school dropout rates are available). Moreover, the school cannot dismiss students if parents stop paying the school fees. Additionally, estimates could be biased if students who otherwise would have enrolled in this particular school did not do so as a result of their father’s job loss. However, given the sample restrictions applied, the students in the analytic sample had to be enrolled in the school before the beginning of the Great Recession.

Moreover, I am implicitly assuming here that school inputs are not altered by parental job loss. This assumption would fail to hold if teacher evaluations differ after father’s job loss. Empirical evidence has shown that evaluations using teacher and externally administered tests differ [see, for instance, Burgess and Greaves (2013) and Gibbons and Chevalier (2008)]. Two of the main reasons that have been put forward to explain this divergence are teacher bias (or statistical discrimination/stereotypes) and information (teacher assessments are based on longer observation, whereas tests evaluate the performance on a specific day). The important question for the consistency of $\rho$ is whether teacher biases could potentially affect the estimates. On the one hand, if teacher bias is fixed, this would be captured by $\alpha _{i}$. On the contrary, estimates of $\rho$ would be biased if teacher’s perceptions of academic performance change when the father loses his job. Existing evidence does not seem to be very helpful in reaching a consensus regarding the direction of the potential bias. Gibbons and Chevalier (2008) find that teacher assessments are upward biased in favour of low-achieving students, whereas Burgess and Greaves (2013) and Hanna and Linden (2012) find evidence that teachers discriminate against minorities in the UK and India, respectively. Unfortunately, there are no data to rule out negative teacher biases towards those students affected by paternal job loss as a potential driver of the negative results found in Sect. 5. However, it seems unlikely that teachers would start evaluating more negatively those students coming from families that are struggling due to the Great Recession. If the contrary is true and teachers favour (in terms of grades) those students affected by father’s job loss, then estimates of $\rho$ would be downward biased in absolute terms.

Additionally, estimates in this paper could be downward bias if the school performance in the control group is negatively affected by the recession. Even if students in the control group are not exposed to parental job loss during the period under analysis, their parents could have suffered wage cuts or, in general, feel a higher degree of job insecurity.^22^

For the sake of comparability with previous studies, and in order to emphasise the importance of being able to use panel data to address the question posed in this paper, the main table in the next section will show results when different identification strategies are followed. In particular, I will compare the fixed effect estimates with the results when the cross section of 2012 is used. This model is similar to the one used in studies that use cross-sectional data and job losses coming from plant closures. The model estimated in this case is as follows:

4 $$\begin{aligned} Y_{i,2012}= \alpha + \rho D_{i} + X'_{i}\beta + \epsilon _{i} \end{aligned}$$

where $X'_{i}$ includes dummies for the stage of education in 2012, the student’s gender and quarter of birth, and a dummy for whether the father has a level of maximum education reached that goes beyond high school. I will also compare the main fixed effects results with those of value-added estimations. The value-added model uses the cross section of 2012 and controls for grades in 2008:

5 $$\begin{aligned} Y_{i,2012}= \alpha + \rho D_{i} + \psi Y_{i,2008} + X'_{i}\beta + \epsilon _{i} \end{aligned}$$

For the impact to be causal, the latter two strategies need to assume that job losses during the Great Recession are exogenous, whereas the main strategy in this paper only needs to rely on a conditional exogeneity assumption, i.e. conditional on worker fixed effects, job losses during the Great Recession are exogenous.

**Table 5:** Effect of father’s job loss on the average grade

Dependent variable:	C.1	C.2	C.3	C.4	C.5	C.6	C.7
Average grade	FE	CS	VA	FE	CS	VA	FE-All
FJL	$-$ 0.147**	$-$ 0.248*	$-$ 0.261***	$-$ 0.133**	$-$ 0.266*	$-$ 0.240**	$-$ 0.081
	(0.062)	(0.141)	(0.084)	(0.063)	(0.151)	(0.102)	(0.061)
Average grade 2008			0.794***			0.941***	
			(0.041)			(0.064)	
Observations	890	178	178	780	178	123	1360
Students	178	178	178	178	178	123	332
Kinder excluded				$\checkmark$	$\checkmark$	$\checkmark$	
Student fixed effects	$\checkmark$			$\checkmark$			$\checkmark$

FJL (father’s job loss): dummy equal to 1 from the year the father loses the job. Average grade scaled to mean 0 with $SD = 1$ based on entire population of 358 students. *, **, *** denote significance at the 10%, 5% and 1% levels. Clustered robust standard errors at the family level in parentheses. There are 137 clusters (35 of them treated). All models include year and stage of education dummies. Except in Columns 1, 4 and 7, regressions include a dummy for father’s education, gender and the student’s quarter of birth. FE: fixed effects, CS: cross section of 2012, and VA: value-added regressions

## Results

### The impact of father’s job loss on children’s school performance

Table 5 presents different estimates of the effect of father’s job loss on the standardised average grade. Standard errors are clustered at the family level. There are 137 clusters in the main analytic sample. The analysis uses the average grade standardised based on the mean and standard deviation of all students in the sample, with all the models controlling for year and stage of education dummies. For the 2012 cross section (CS in Table 5, see Eq. 4) and value added (VA in Table 5, see Eq. 5) regressions, standardisation of the average grade variables is performed at the year level.^23^

Using the strict definition of involuntary job loss, the results with the main identification strategy used in this paper are shown in Column 1 (see Eq. 1). Fixed effect estimates show that after father’s job loss, students suffer a decrease in average grades of about 15% of a standard deviation. Columns 2 and 3 use cross-sectional data instead, with additional controls including a dummy that indicates whether the father has an education level beyond high school, the gender and the quarter of birth of the student (not shown). Column 2 shows the results when the cross section of 2012 is used, in a fashion similar to that used in the plant closure literature. The magnitude of the coefficient increases considerably compared to that in Column 1. Controlling for the student’s average grade prior to father’s job loss in the 2012 value-added regression shown in Column 3 increases the precision of the estimate. This value-added result indicates that father’s job loss entails a significant decrease in average school performance that almost doubles the impact measured with the fixed effect regression in Column 1. This exercise suggests that, unless the set of controls available is extremely rich, the two latter strategies render estimates that could suffer from potential bias due to (negative) selection of whom is laid off and underlines the usefulness of panel data to address it.

Given that the information to construct kindergarten grades differs from the information in compulsory education grades, Columns 4 to 6 in Table 5 repeat the same exercise but excluding from the analysis and the standardisation process all those grades obtained in kindergarten. The results are very similar to those obtained in Columns 1 to 3. In order to not disregard additional observations, the remaining analysis will continue to use kindergarten grades. All the previous results in Columns 1 to 6 are based on the restricted sample, i.e. the one resulting after applying the sample restriction criteria outlined in Sect. 3. In Column 7, I present results using the full sample of students observed in 2012. The point estimate in Column 7 is smaller (in absolute terms) than the one in Column 1, but the results point in the same direction. This decrease in the magnitude is expected since this regression includes students that are not observed in 2008, but only after father’s job loss has occurred (and therefore, for these students the grade might had already decreased or started decreasing by the time they entered the sample).

Results go in the same direction when using the other two definitions of job loss (see Table 14 in Appendix B). As it could be expected given the more loose definition of job loss used in Panel A, the magnitude of the estimates decreases with respect to those in Table 5. And vice versa for the results in Panel B: Children of fathers suffering job loss due to downsizing or firm closure suffer a decrease in the average grade of almost 22% of a standard deviation (Panel B, Column 1). The remaining analysis will continue to utilise the strict definition of involuntary job loss used in Table 5.

**Table 6:** Placebo: average effect of father’s job loss on the cross section of 2008

Dependent variable	C.1	C.2
Average grade		
Future FJL	$-$ 0.010	0.013
	(0.132)	(0.158)
Father has high	0.355***	0.580***
education level	(0.133)	(0.162)
Female	0.450***	0.531***
	(0.124)	(0.147)
Born in Q1	0.537***	0.684***
	(0.187)	(0.229)
Born in Q2	0.234	0.355*
	(0.167)	(0.214)
Born in Q3	0.317**	0.490**
	(0.160)	(0.194)
Students	178	123
Kinder excluded		$\checkmark$

Future FJL: equals 1 for those students whose father suffers a job loss in the period under analysis (treated students) after 2008, using the strict definition of job loss. Average grade scaled to have mean 0 with $SD = 1$ based on corresponding 2008 population. *, **, *** denote significance at the 10%, 5% and 1% levels. Clustered robust standard errors at the family level in parentheses. All models include stage of education dummies

**Table 7:** Excluding fathers that in 2008 had a lower labour market attachment

Dependent variable: average grade	C.1	C.2	C.3	C.4
FJL	$-$ 0.203***	$-$ 0.202***	$-$ 0.173**	$-$ 0.161**
	(0.073)	(0.074)	(0.069)	(0.076)
Proportion treated	21.71%	23.24%	22.62%	21.69%
*N*	760	710	840	830
Students	152	142	168	166
Subsample	More than 3 years of tenure	More than 6 years of tenure	Exclude fathers with temporary contract	Exclude fathers with multiple job losses

Fixed effects estimates. FJL (father’s job loss): dummy equal to 1 from the year the father loses the job. Average grade scaled to mean zero with $SD = 1$ based on entire population of 358 students. *, **, *** denote significance at the 10%, 5% and 1% levels. Clustered robust standard errors at the family level in parentheses. All models include year dummies and dummies for the stage of education. Except for C.4, all the sample restrictions are based on the 2008 data. Strict definition of job loss used

Tables 6 and 7 suggest that the effects found in Table 5 for the fixed effect regressions are likely to be of a causal nature. In Column 1 in Table 6, I show the results of a placebo test using the strict definition of job loss. Future job losses (i.e. job losses that will happen later in the period) should not have an impact on the average grade of students prior to father’s job loss in 2008 (the first academic year in the sample, where by construction all students have employed fathers). The estimate for the main variable of interest is highly imprecise and very close to zero. This finding provides evidence against the possibility that changes in household’s unobservables simultaneously drive father’s job loss (FJL) and school performance of their offspring, since otherwise we would expect to see significantly worse school performance prior to father’s job loss. I additionally display the coefficients of different control variables in Column 1, in order to show that the average grade measure is sensible even in this rather small sample. The results are in line with facts well established in the economics of education literature: students coming from better socio-economic backgrounds (as measured by the father’s education level) perform better and females obtain better grades as well as those born at the beginning of the year (the oldest in the class). Results in Column 2 confirm that the results are the same when kindergarten grades are excluded from the analysis.

The former placebo test indicates that future paternal job losses do not significantly affect grades prior to father’s job loss. This evidence does not guarantee that the grades of treated students were already suffering a decline prior to father’s job loss. However, the evidence shown in Sect. 4 does, given that Fig. 4 showed that there are no existing negative trends in school performance for treated students prior to treatment.

Another way to partially address this issue is to check whether the impact of father’s job loss is mainly driven by those students whose fathers had a lower labour market attachment prior to job loss. One potential way of defining labour market attachment is to use the information on years of tenure at the firm before losing the job. Workers with lower tenure prior to job loss might have been on a different (negative) trajectory prior to losing their job during the Great Recession, and this could, in turn, have affected the performance of their offspring. In order to verify this, I consider only those students whose fathers in 2008 had at least three (or six) years of tenure in their jobs, respectively. Results are shown in Column 1 (and 2) of Table 7. The estimates show that the impact of FJL remains negative and significant in all cases. That is, the negative effect of FJL does not seem to be driven by those students whose fathers had lower tenure at the firm prior to job loss, but rather, by those students whose fathers had a more stable situation prior to losing the job. This is in line with several papers in the job loss literature that have found that workers with longer tenure prior to losing their jobs suffer more after job loss in terms of income declines and employment probabilities.^24^ In this sense, these children would suffer a larger shock after paternal job loss than those children whose fathers had, prior to job loss, a lower labour market attachment. In Column 3, I use an alternative definition for labour market attachment based on the type of contract the father had prior to job loss: students whose fathers had a temporary contract in 2008 are excluded from the sample. The results are very similar to those shown in Columns 1 and 2. Additionally, suffering multiple job losses during the period under analysis might also indicate a rather unstable attachment to the labour market. However, it is also possible that multiple job losses indicate a better ability to find new employment during the Great Recession. In any case, multiple job losses could be due to unobserved time varying heterogeneity that could bias the estimates. In Column 4, I exclude students whose fathers have experienced more than one job loss in the period. Stevens (1997) studied the effects of multiple job losses on earnings and found that much of the persistence in the earnings losses can be explained by additional job losses in the years following an initial displacement. Initial displacements predict future displacements, and thus, subsequent displacements might not be exogenous (in the sense that they might no longer be attributed to the combination of the Great Recession and fixed effects). By excluding from the sample those students whose fathers experienced multiple job losses during the period under analysis, the estimate remains negative and very similar to the other point estimates in Table 7. All in all, the evidence in Tables 6 and 7, together with Fig. 4, suggests that treated students were not on a different (negative) trend prior to father’s job loss.

**Table 8:** Robustness check: group-and-year and group-and-stage of education specific effects

Dependend var: Average grade	C.1	C.2	C.3	C.4	C.5	C.6	C.7
*Panel A. Group-and-year specific effects*							
FJL	$-$ 0.144**	$-$ 0.131**	$-$ 0.143**	$-$ 0.145**	$-$ 0.153**	$-$ 0.144**	$-$ 0.132**
	(0.061)	(0.061)	(0.061)	(0.066)	(0.061)	(0.062)	(0.064)
*Panel B. Group-and-stage of education specific effects*							
FJL	$-$ 0.147**	$-$ 0.117*	$-$ 0.148**	$-$ 0.149**	$-$ 0.146**	$-$ 0.150**	$-$ 0.123**
	(0.061)	(0.059)	(0.061)	(0.061)	(0.061)	(0.061)	(0.057)
*N*	890	890	890	890	890	890	890
Students	178	178	178	178	178	178	178
Group specific trends ($\delta _{tj})$	Father’s education	Father’s income	Father owned business	Father’s sector	Mortgage or rent	Student’s gender	All previous

Fixed effects estimates. FJL (father’s job loss): dummy equal to 1 from the year the father loses the job. Average grade scaled to mean zero with $SD = 1$ based on entire population of 358 students. *, **, *** denote significance at the 10%, 5% and 1% levels. Clustered robust standard errors at the family level in parentheses. All models include year and stage of education dummies. All variables interacted with the year or stage of education dummies are measured in 2008, except the mortgage/rent indicator which is measured in 2012 due to lack of data in 2008. Strict definition of job loss used

A further potential concern raised in Sect. 4 is that estimates might be driven by negative trends in school performance for particular groups of students, either because of differential effects by subgroups as the crisis unfolded, or due to differential trends in parental investment as the child advances within the education system. To address these concerns, I show in Table 8 the results of different regressions where the original model is augmented by interacting the year dummies (stage of education dummies) with certain group-specific characteristics measured prior to job loss in Panel A (B). The year (stage of education) dummies are interacted with a variable that is equal to 1 if the father has a high level of education (beyond high school) in Column 1; a variable that equals 1 if the father was classified in the high-income category in 2008 (Column 2) and a dummy that equals 1 if the father owned a business in 2008 (as opposed to working for a firm) in Column 3. The year (stage of education) dummies are also interacted with dummies for the sector the father was employed in 2008 (3 sector categories are used: manufacturing, construction and services) in Column 4, whether the household lives in a house that is fully paid in Column 5 and student’s gender in Column 6.^25^ The last model (Column 7) includes all the group specific trends in Columns 1 to 6 together. The point estimates shown in Table 8 are all very similar to those in Column 1, Table 5. This evidence suggests that the estimates presented so far do not simply reflect differential group-and-year specific effects or group-and-stage of education effects.

**Table 9:** Impact of mother’s job loss (and labour market status) on school performance

Dep var: Average grade	C.1	C.2	C.3
MJL	$-$ 0.071	$-$ 0.035	
	(0.082)	(0.081)	
FJL		$-$ 0.142**	$-$ 0.140**
		(0.064)	(0.061)
Mother works			0.126
			(0.078)
Obs	835	890	889
Students	167	178	178
Subsample	Mother employed in 2008	All restricted sample	All restricted sample

Fixed effects estimates. FJL/MJL (father’s/mothers job loss): dummy equal to 1 from the year the father loses the job. Mother works is a dummy that equals 1 in the years the mother is observed working. Average grade scaled to mean zero with $SD = 1$ based on entire population of 358 students. *, **, *** denote significance at the 10%, 5% and 1% levels. Clustered robust standard errors are at the family level in parentheses. All models include year dummies and dummies for the stage of education. Strict definition of job loss used

### The role of mother’s job loss

In Column 1, Table 9, I show the impact of mother’s job loss (MJL) on her children’s school performance. The same analytic sample used so far is employed here, but further excluding those mothers that were unemployed at the beginning of the period. The MJL variable is defined in the same way as FJL, using the preferred definition of strict job loss. It is equal to 1 from the (academic) year that the mother loses her job. The results in Column 1 show that there is a negative but no significant effect of MJL, on average, on her children’s school performance. These results do not seem to be driven by the fact that women in a country like Spain might have a lower labour market attachment. As it was shown in Table 2, 81% of the mothers in the analytic sample were employed in 2008.

This paper differs from almost all other papers in the literature in the sense that job losses happen during a deep economic crisis. If mother and father job losses are correlated (which is indeed the case in this sample), then the effect of FJL could also be capturing the impact of MJL on the average grades of their offspring. The results in Column 2 suggest that this is not the case. The MJL coefficient becomes smaller in magnitude when father’s job losses are introduced, whereas the FJL coefficient remains almost the same (see Column 1, Table 5). Finally, mothers could react to father’s job loss by going back to work (in the case that they were unemployed prior to FJL). The results for the FJL variable shown in Column 3 barely change when augmenting the specification with a dummy variable that equals 1 whenever the mother is employed, and 0 otherwise.

The findings in this section could partly be explained by the results in recent papers in health economics that have found that men suffer more negative health-related consequences after job loss than women. For instance, Kuhn et al. (2009) find that job loss significantly increases expenditures for antidepressants and related drugs, as well as hospitalizations due to mental health problems for men, but not for women. Eliason and Storrie (2009a) find that job loss produces a twofold short-run increase in suicides and alcohol-related mortality for both sexes. However, overall mortality risk among men increased by 44 percent during the first 4 years following job loss while there was no impact in the longer run or on female overall mortality. Eliason and Storrie (2009b) find that job loss significantly increases the risk of hospitalisation due to alcohol-related conditions, among both men and women, and due to traffic accidents and self-harm among men only.

In terms of earnings decline after job loss, both men and women suffer substantial decreases in the probability of being observed in the high-income category (a decline of 31% and 24% for men and women, respectively). The larger contribution of fathers to household income could also be behind these results. Whereas 65% of the fathers were observed in the high-income category in 2008, only 24% of mothers reported to be in the high-income category. Findings reported by social psychologists suggest that there are detrimental effects of job insecurity (something that is likely to be positively related to job loss) on financial anxiety for men but not for women (Lim and Sng 2006).

A further potential explanation could be found in the theories of social roles and identity, as pointed out by Rege et al. (2011) who come to similar conclusions as this paper with regard to the effects of maternal and paternal job loss. These authors highlight that social norms and historical employment patterns have allowed women to develop a greater range of non-employment-related roles. This, in turn, would make women more adaptable and equipped to handle job loss, whereas job loss could be more detrimental for men because a large part of their identity is connected to their specific job.

### Alternative treatment definitions and the role of long-term unemployment

So far, given the reasons stated in Sect. 4, the treatment variable has been defined as an absorbing state (i.e. it equals 1 from the moment the father loses the job, irrespective of his employment situation afterwards). However, it is interesting to see what happens if the treatment definition is changed to allow those fathers who find a job after job loss to switch treatment status.

**Table 10:** Alternative treatment definitions and the role of long-term unemployment

Dependent variable: average grade	C.1	C.2
All FJL	$-$ 0.082	
	(0.052)	
FJL leading to long-term unemployment		$-$ 0.237**
		(0.106)

*N* $=$ 890 observations; 178 students. Fixed effects estimates. All FJL: dummy equal to 1 the year the father loses the job and the years after job loss as long as the father remains unemployed. FJL leading to long-term unemployment: dummy equal to 1 the year the father loses the job (as long as he does not find a job the same year), and the years after job loss as long as the father remains unemployed. Average grade scaled to mean zero with $SD = 1$ based on entire population of 358 students. *, **, *** denote significance at the 10%, 5% and 1% levels. Clustered robust standard errors at the family level in parentheses. All models include year dummies and dummies for the stage of education. Strict definition of job loss used

I show the results of experimenting with two different treatment definitions in Columns 1 and 2 in Table 10. ‘All FJL’ is a dummy variable that measures the impact of all unemployment spells irrespective of the duration, whereas ‘FJL leading to long-term unemployment’ only equals 1 if (and when) the father stays unemployed at least for an academic year.^26^ In this sense, ‘FJL leading to long-term unemployment’ would be capturing the effect of long-term unemployment spells, whereas ‘All FJL’ would be capturing the impact of father’s job loss and long-term unemployment. The results in Columns 1 and 2 of Table 10 suggest that the negative impact of father’s job loss on the average grade is mainly driven by those fathers that stay unemployed for at least one academic year.

### Additional robustness checks

I present a series of additional robustness checks in Table 11. In Column 1, I only use information from academic years 2008, 2010 and 2012. As described in Sect. 3, by restricting the sample to these periods I do not need to make any assumptions with regard to the exact date of job loss. The estimates in Column 1 show that the coefficients of the FJL variable are also negative and significant, and slightly larger in magnitude. In Column 2, I present results when controlling for grade-specific dummies (rather than stage of education dummies). Results are very similar to the preferred estimate. Given the small sample size, it is important to verify that outliers are not the main drivers of the results. In order to address this concern, in Column 3 I show the FJL estimate when observations at the extremes of the grade distribution are dropped. I calculate the average change in the average grade between the academic years 2008 and 2012 and run the main specification excluding observations for which the average change falls in the 5th and 95th percentile. Applying these restrictions has almost no effect on the estimate of FJL.

**Table 11:** Other robustness checks

Dependent variable: average grade	C.1	C.2	C.3	C.4	C.5	C.6
FJL	$-$ 0.189**	$-$ 0.126*	$-$ 0.113*	$-$ 0.131**	$-$ 0.124*	
	(0.075)	(0.064)	(0.058)	(0.064)	(0.064)	
% FJL in grade-year				$-$ 0.004		
				(0.004)		
% FJL in grade-year-class					$-$ 0.001	
					(0.001)	
JL main earner						$-$ 0.125**
						(0.060)
*N*	534	890	800	890	882	1000
Students	178	178	160	178	178	200
Robustness check	Using only 2008–2010–2012	Controlling for grade	Outliers: Excl 5th/95th perc	Peer effects in same grade	Peer effects in same class	Job losses main earner

Fixed effects estimates. FJL (father’s job loss): dummy equal to 1 from the year the father loses the job. Average grade scaled to mean zero with $SD = 1$ based on entire population of 358 students. *, **, *** denote significance at the 10%, 5% and 1% levels. Clustered robust standard errors at the family level in parentheses. All models include year dummies and stage of education dummies (except in C2). Strict definition of job loss used

Job losses in this article happen during a period where many individuals lost their jobs. In order to understand whether general job losses are affecting the results, I calculate the percent of students whose fathers suffered a job loss in the same grade and year, and also in the same grade, year and class. This controls for potential peer effects of parental job losses of classmates/friends. Introducing these variables in the main specification (see Columns 4 and 5) barely changes the original point estimates of FJL in the preferred specification in Table 5 (Column 1). Moreover, the peer group effect coefficients are not significant. Finally, Column 6 examines the role of job loss of the main earner in the household. As pointed out in Sect. 3, if parental job loss leads to separation or divorce and the father moves out during the period under analysis, the sample will not register these cases. Therefore, the results shown so far are constrained to the sample of students whose parents have stayed together during the period under analysis. In Column 6, *JL main earner* is defined in the same way as the FJL variable, but taking into account the job losses of the mother when the father is not present in the household. The results barely change.

**Table 12:** Heterogeneous effects

Dependent variable: average grade	C.1	C.2	C.3	C.4	C.5	C.6
FJL	$-$ 0.127*	$-$ 0.187**	$-$ 0.240**	$-$ 0.152*	$-$ 0.229***	$-$ 0.340***
	(0.071)	(0.088)	(0.092)	(0.087)	(0.079)	(0.097)
FJL*Father’s educ beyond HS		0.201*				
		(0.121)				
FJL*Father worked in a firm in 2008			0.166			
			(0.121)			
FJL*Older students (in secondary 2012)				0.057		
				(0.110)		
FJL*owning house in 2012					0.249**	
					(0.118)	
FJL*household not moving in the period						0.246**
						(0.117)
*N*	890	890	890	890	890	880
Students	178	178	178	178	178	176
*P* value (FJL 1 $=$ FJL 2)		0.099	0.174	0.604	0.037	0.037

Fixed effects estimates. FJL (father’s job loss): dummy equal to 1 from the year the father loses the job. Average grade scaled to mean zero with $SD = 1$ based on entire population of 358 students. *, **, *** denote significance at the 10%, 5% and 1% levels. Clustered robust standard errors at the family level in parentheses. All models include year and stage of education dummies. All specifications use the strict definition of job loss. *P* value (FJL 1 = FJL 2) is the *P* value coming from a test to analyse whether the coefficients of the two subgroups compared in each column are equal

### Heterogeneous effects

Following the related literature, I analyse whether the impact of father’s job loss is heterogeneous across different subgroups in Table 12. The results need to be interpreted with caution given the limitation posed by the sample size (standard errors tend to be large), but nevertheless they add new suggestive evidence on likely mechanisms. In Column 1, I reproduce the results of the preferred specification.^27^ The subsequent models in the table interact the FJL variable with one characteristic at a time. The results suggest that the effects of FJL are concentrated on those students whose fathers have low levels of education (in Column 2, Table 12, the *P* value for the interaction is just under the 10%).^28^^,^^29^ The results also suggest that those students whose fathers lost their jobs because they closed their businesses suffer a more detrimental effect of FJL (Column 3). However, standard errors for the interaction term are too large to draw any strong conclusions. Additionally, FJL does not seem to have a differential impact for older students in Column 4 (older students are those enrolled in secondary education in 2012). Column 5 shows that the effect of FJL is mainly concentrated on those students whose families either rent or have a mortgage (as opposed to households that fully own their property).^30^ Finally, the results in Column 6 show that the negative effect of FJL seems to be more detrimental for those students whose families moved during the period. The results in the last two columns might be picking up the same mechanism, if families that suffered FJL and were not in possession of a fully paid property, were evicted or had to move as a consequence of FJL.

**Table 13:** Income reductions across different subgroups

	C.1	C.2	C.3	C.4	C.5	C.6
Dependent variable: Father’s income (=1 if father has high income)						
FJL	$-$ 0.318***	$-$ 0.354***	$-$ 0.332*	$-$ 0.366***	$-$ 0.470***	$-$ 0.553*
	(0.099)	(0.117)	(0.181)	(0.133)	(0.135)	(0.308)
FJL*Father’s educ beyond HS		0.111				
		(0.210)				
FJL*Father owned business in 2008			0.021			
			(0.214)			
FJL*Older students (in secondary 2012)				0.103		
				(0.148)		
FJL*owning house in 2012					0.342**	
					(0.171)	
FJL*household not moving in the period						0.248
						(0.325)
*N*	829	829	829	829	829	819
Students	169	169	169	169	169	167
*P* value (FJL 1 = FJL 2)		0.598	0.922	0.489	0.047	0.447

Fixed effects estimates. High income is defined as having monthly net income in the 2 highest categories in the survey (more than 1500 euro net). FJL (father’s job loss): dummy equal to 1 from the year the father loses the job. *, **, *** denote significance at the 10%, 5% and 1% levels. Clustered robust standard errors at the family level in parentheses. All models include year dummies. Missing observations due to missing values in the income variable (additional missing values in C.6 related to moving information). All specifications use the loose definition of job loss. *P* value (FJL 1 = FJL 2) is the *P* value coming from a test to analyse whether the coefficients of the two subgroups compared in each column are equal

Several papers in the literature have documented a considerable reduction in earnings after job loss. For instance, Jacobson et al. (1993) reported that high-tenure workers separating from distressed firms suffer long-term losses averaging 25% per year. Accordingly, Column 1 in Table 13 shows that after job loss, the fathers in the sample are about 32 percentage points less likely to be observed in the high-income category. The remaining models in Table 13 explore whether the heterogeneous effects shown in Table 12 could be due to differential reductions in income by group. This is done by regressing the FJL variable on a dummy variable that equals 1 if the father is observed in the high-income category, controlling for individual fixed effects. The order of the columns is the same as in Table 12. The evidence partly suggests that those groups with a smaller decrease in average grades are also those groups with a smaller decrease in income, although standard errors of the FJL variable are rather large and the coefficients on the interactions are highly insignificant in most cases. The different income reductions across groups could be suggesting that income is one of the mechanisms driving the negative effect of paternal job loss on the school performance of their offspring. However, it is important to note that these results are obtained for students who are enrolled in the same school during the five periods of observation. The observed reduction in income cannot be linked, therefore, to changes in the school attended after job loss. Reductions in income could partly explain the results if fathers decrease hours of extra help with homework (or other extra school activities) after job loss or decrease consumption that could be related to school performance. Additionally, income reductions after father’s job loss could entail higher stress or financial anxiety and uncertainty for affected individuals and households, as some papers in social psychology and health economics have suggested [see, for instance, Lim and Sng (2006) and Kuhn et al. (2009)].

## Conclusion

The available evidence on the effects of job loss has shown that affected individuals suffer important earning losses when re-employed, deteriorated physical and mental health and an increase in the likelihood of getting a divorce, among other negative consequences. These negative consequences have a direct effect on some of the inputs that are generally seen as affecting the production function for cognitive achievement. Accordingly, several studies have shown that parental job loss has a negative impact on children’s educational outcomes. In doing so, they have used plant closures or local labour market conditions as a way to circumvent the endogeneity of parental job loss (see Sect. 2).

As Kalil (2013) noted in her review of the effects of the Great Recession on child development, we know very little about the Great Recession’s impacts on children. This paper has contributed to this literature by looking at the intergenerational impact of labour market shocks (parental job loss) on school performance during the Great Recession in Spain. As Rege et al. (2011) point out, estimating a causal relationship between parental job loss and child outcomes faces two main challenges: concerns of omitted variables (and therefore the need to find a source of exogenous variation for parental job loss) and the scarcity of appropriate data. This paper has addressed both of these concerns by exploiting job losses due to the Great Recession in Spain and by using fixed effect models that allow exploitation of within individual variation in school performance. The results using the fixed effect model have been compared to those obtained in a fashion similar to that used in the plant closure literature, and those coming from value-added regressions. The estimates coming from the two latter strategies were considerably larger in magnitude. This suggests that if the data are not sufficiently rich to control for potential determinants of school performance linked to parental job loss, these strategies might be rendering estimates that could suffer from potential selection bias due to whom is laid off.

The results in this paper imply that father’s job loss entails an average decrease in children’s average grades of about 15–22% of a standard deviation, although the effects are larger for particular subgroups. Compared to Rege et al. (2011), who find an average effect of father’s plant closure on average GPA of 16-year-olds of about 6% of a standard deviation, the results here suggest that the effects of father’s job loss on the average grade of their offspring during a deep economic crisis are larger in magnitude (although Rege et al. (2011) main estimate is within this paper’s main estimate confidence interval). Nonetheless, as Currie et al. (2015) note, these larger estimates are consistent with the fact that the Great Recession represented a massive and unprecedented economic shock for millions of families, which could potentially be more long-lasting than the shock induced by job losses due to plant closures.^31^ Rege et al. (2011) compare their estimates with the results summarised by Hanushek (2006) about the STAR experiment. Hanushek notes that large class size reductions of around 8 students are necessary in order to increase students’ achievement by 20% of the standard deviation. Thus, the effects of father’s job loss on the school performance of their offspring during the economic recession in Spain are quite sizeable.

Given the panel nature of the data set used, I have performed placebo tests that show that school performance prior to father’s job loss is not affected by future job losses. Additionally, the negative effect of paternal job loss is not driven by those students whose fathers had a lower labour market attachment prior to job loss. On the contrary, and in line with Jacobson et al. (1993), the negative impact of father’s job loss seems to be driven by those fathers who had a more stable labour market situation prior to losing their jobs. This evidence suggests that treated students were not on a different (negative) trend prior to father’s job loss. Moreover, the average grade does not exhibit a negative trend prior to treatment, and the results are robust to the inclusion of differential group-year (group-and-stage of education) effects.

The average impact of mother’s job loss on school performance is close to zero and non-significant. These results are in line with those reported by Rege et al. (2011). They argue that the disparate effect of job loss across fathers and mothers is consistent with recent empirical studies documenting that the mental distress experienced by displaced workers is generally more severe for men than women. Moreover, the average results found in this paper mask important differential impacts across subgroups. The negative impact of father’s job loss on school performance is mainly driven by those fathers who suffer longer unemployment spells. Related to this, the effect of father’s job loss appears to be largely concentrated among children of already disadvantaged families in terms of the educational level of the father.

Given the massive employment destruction that took place in several advanced economies during the past Great Recession, and more recently during the COVID-19 crisis, the present study underlines the importance of understanding the mechanisms behind the negative and sizeable effect of father’s job loss on children’s school performance. Moreover, this article has looked at the short-term impact of parental job loss on school performance. As data become available, future research should look at more long-term effects: Does parental job loss leave permanent scars on individuals? In particular, does parental job loss during childhood affect later educational and labour market outcomes? Besides the importance of the question in terms of granting equality of opportunity to individuals in society, there are also important implications for the economy as a whole, given the paramount importance of human capital for economic growth.

## Figures and Tables

**Fig. 5:**
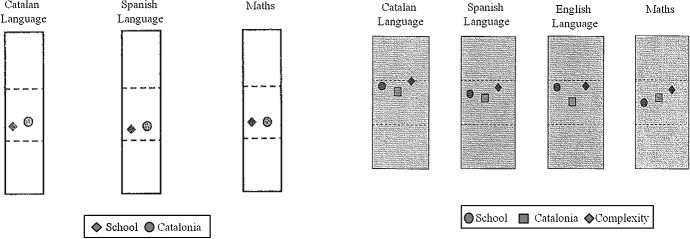
Position of the school in the Catalan Education system *Source*: Primary school evaluation, 2008–2009 (left panel) and 2011–2012 (right panel). Education Department, Generalitat de Catalunya (Catalan Government)

**Fig. 6:**
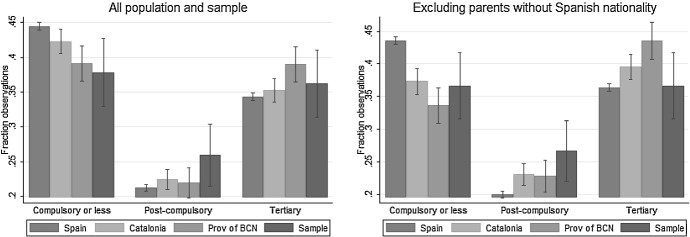
Father’s education. Mean (given by the height of the bar) and 95% confidence intervals. Population data refer to the first quarter of 2012 of the Spanish LFS. Weights have been used for the LFS data

**Fig. 7:**
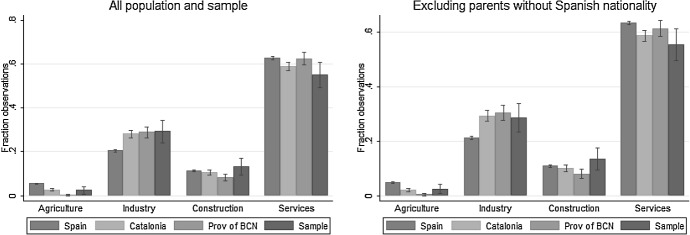
Father’s sector of the firm. Mean (given by the height of the bar) and 95% confidence intervals. Population data refer to the first quarter of 2012 of the Spanish LFS. Weights have been used for the LFS data

**Fig. 8:**
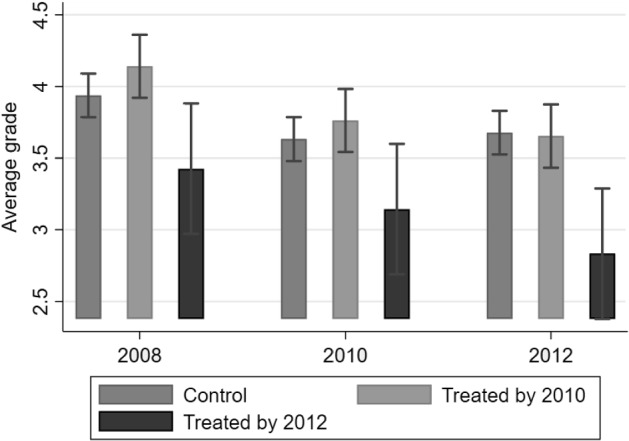
Average grade in 2008, 2010 and 2012 by treatment status. *Note*: Mean (given by the height of the bar) and 95% confidence intervals. The average grade in a given academic year goes from 1 to 5 (where 1 means that the student has failed the subject and 5 is the best possible grade). The sample used is the one after applying the restrictions explained in the main text. Looser definition of job loss used here to define treated students, to give an overview of all students whose fathers lose their jobs during the period under analysis. Results are very similar using the strict definition of job loss

## A1 Representativeness

### A1.1 The school within the Catalan school system

The school where the data have been collected is a concerted school in the province of Barcelona. A concerted school is a private school, typically owned by the Catholic Church (80% of them are including this particular school), that signs a long-term concert or agreement with the government by which it becomes fully subsidised in exchange for implementing a state school-like admission policy (Arellano and Zamarro 2007). Neither public nor concerted schools, in principle, charge fees because they are both funded by the taxpayers. However, in practice the cost of attending concerted schools may be three times larger than for state schools, but in either case these are much smaller amounts than those faced by the small fraction of parents that send their children to non-concerted fee-paying private schools (Arellano and Zamarro 2007). For this particular school, the fees vary according to parental income, but for kindergarten and compulsory schooling ages, it is below or around 100 euros per month.

Figure 5 provides a description of the position of the school in the Catalan education system in terms of the achievement of its students. The information comes from a test designed and organised by the Education Department of the Catalan Government that all 6th graders (primary school) must take regardless of school type. The first test was administered in the academic year 2008–2009 and evaluated core competencies in Catalan, Spanish and mathematics. From the next academic year, it also tested the English knowledge of 6th graders. The results of these tests are not publicly available, but the schools receive a document with information about how their students performed compared to the average school in Catalonia, and this is what is shown in Fig. 5 for the academic years 2008–2009 and 2011–2012.

The results divide the level of core competencies into three categories: low (0–70), average (70–90) and high (90–100). In 2008–2009, the results for 6th graders in the school where the data have been collected are very close to the average results in Catalonia (for the three tested subjects). The same can be stated about the academic year 2011–2012 on average, although the students in the school analysed here performed slightly worse in English and slightly better in mathematics. The school does not have the data needed to compute whether the differences between the school and the Catalan average results are significantly different from each other. Nevertheless, these results seem to suggest that, in terms of academic results, the school is very close to the average Catalan school.

### A1.2 The characteristics of the sample compared to the population in the Spanish Labour Force Survey

I use the data of the Spanish Labour Force Survey (LFS) to assess how the distribution of some key characteristics of the individuals in the sample compares to the distribution of these same characteristics in the Spanish, Catalan and (province of) Barcelona population.

I use the LFS data produced by the Spanish National Institute of Statistics (INE) corresponding to the 1st quarter of 2012 and extract the subsample of individuals aged 0–20. Since the age data are given in 5 year interval age groups, and given that the individuals in the school have ages ranging from 3 to 18, this is the closest I can get to the ages in the sample. Using the information on family relationships, I have matched these individuals with the information of their parents.

The distribution of father’s education, father’s labour market status and father’s sector of activity are shown in Fig. 2 (in the main text) and in Figs. 6 and 7. In general, there is a higher fraction of fathers in the sample with post-compulsory (non-tertiary) education if compared to any of the three different populations extracted from the LFS, and whether I use all the population (left figure) or I restrict the sample to include only children from Spanish citizens (right figure). Nonetheless, the results of Pearson square tests indicate that the frequency distribution of education levels for fathers in the sample resembles that of fathers in Catalonia and Barcelona when the whole population is considered and that of Catalonia when the sample is restricted to Spanish nationals. Similar figures for the distribution of the labour market status of fathers in the sample and Spain, Catalonia and the province of Barcelona are shown in the main text. For those fathers that are working, the distribution regarding the sector of activity is plotted in Fig. 7. Both for the whole population and for the restricted one, the frequency distribution of the sectors in the sample resembles that of Catalonia.^32^

The descriptive analysis in this section suggests that the sample, even if restricted in terms of size and concentrated in only one school, is sufficiently diverse to reproduce some of the most representative characteristics of the population with children aged 0–20 (as given by the data in the Spanish LFS).

### A1.3 How representative is the sample of the school population?

The school granted access to the data that they had in electronic format for the population of all children enrolled at the school during the academic year 2011–2012. In particular, I got access to data related to the distribution of students by grades, their birth dates and the postcodes associated with the household residence for all students enrolled at the school during the academic year 2011–2012. In general, there are no remarkable differences between the sample and the school population regarding neighbourhood, month of birth and distribution of the students across different grades (cohorts). The results of the Pearson Chi-square tests indicate that there are no significant differences between the frequency distribution in the sample and the school population for any of these variables. I also got access to data on school outcomes for the school population. Unfortunately, these data were not available in electronic format for all the school population, but just for some groups (e.g. for some subjects, grades and classes). Therefore, when assessing representativeness using outcome data, I use the data of those students enrolled in grades for which I also observe the final outcomes of the school population. The data for both the sample and the school population refer to the third term of the academic year 2011–2012. For both mathematics and Catalan, there are not significant differences between the frequency distribution in the sample and the school population for both primary and secondary school students with Spanish-like surnames (if taking into account the whole sample, students in the sample tend to perform better, but this is no longer the case when students without Spanish-like surnames are excluded from both the sample and the population).^33^

Unfortunately, the data available for the whole population of students in the school in 2012 do not allow me to discern respondents from-non respondents. I can therefore not analyse whether some of the available characteristics (postcode, month of birth, etc.) play an important role in the decision to fill out the questionnaire. However, the previous comparison of the available data for the school population with the sample data seems to suggest that the sample is representative of the school population as long as students with an immigrant background are excluded from both the sample and school population.

## A2 Survey and item non-response

There were 931 children (distributed over 700 families) enrolled at the school where the data were collected during the academic year 2011–2012. Some children were not present at the school the day the questionnaires were handed out, but we were able to distribute the questionnaires to children in 630 families. A total of 313 families returned the questionnaires. Since there could be more than one child enrolled at the school for each family, these data corresponded to 436 children distributed throughout all the grades. The response rate is, therefore, close to 50% of the questionnaires delivered. After receiving, coding and revising the questionnaires, three typologies of answers emerged. First, a total of 242 families returned the questionnaires completed (there were some minor cases of item non-response, but in general the answers were complete and consistent). Second, some inconsistency was found for the answers of either the father or the mother in 58 families. In order to correct these inconsistencies, the school allowed me to contact those 58 families again. Twenty-one of those families returned the questionnaire corrected. Finally, out of the 313 returned questionnaires, 13 of them presented a substantial number of questions unanswered. These 13 families have been disregarded from the analysis. Additionally, 8 families did not provide the name of the children in the returned questionnaires, and therefore, it was impossible to match the parental data provided with their children’s grades. Taking this into account, the final sample consists of 408 students in 292 families (358 of them were enrolled in compulsory education grades in 2012). Item non-response is very low for almost all questions in the household and individual questionnaires. Importantly, there is almost no missing data regarding the labour market status of the father or the mother after following up with respondents to correct mistakes.

## A3 The data on school outcomes

The different stages in the Spanish education system are as follows. Kindergarten is divided into a first stage (children from ages 0–3) and a second stage (children from 3 to 6 years of age). Even if kindergarten is not compulsory, 98% of 3-year-olds were enrolled in the second stage of kindergarten in the academic year 2008.^34^ Compulsory education in Spain begins the year the child turns six until the year the child turns sixteen, and is divided into two stages: primary school, with a total of six grades (until the year the child turns twelve) and secondary school, comprising four additional grades. After completing compulsory education successfully, students can choose to enrol in high school for two additional years or in vocational training.

Grades’ formats differ across stages of education. In the school where the data come from, grades in secondary school have a number format ranging from 1 to 10, with 10 being the best possible grade and the passing grade being bigger or equal to 5. In primary school, grades can take on five different values: (1) fail, (2) pass, (3) good, (4) very good and (5) excellent. Secondary school grades can be translated into this five value scale following the traditional convention in the school: grades 1 to 4 in secondary school were assigned a grade of fail (1). A grade of 5 or 6 in secondary school corresponds to pass (2) or good (3), respectively. Grades 7 or 8 correspond to very good (4). Finally, grades 9 or 10 are translated into a grade of excellent (5). In the school analysed, the student in primary, secondary or high school receives a report with her grades three times during each academic year. In the second stage of kindergarten, parents receive a report twice a year where different areas (mathematics, language, arts and musical education) are evaluated with short sentences. These short sentences can be clearly positive (ex: the child can count from one to five, the child can write her name, etc.), clearly negative (ex: the child cannot count from one to five, the child cannot write her name, etc.), or improving type of sentences (child’s counting has improved, etc.). In order to translate these sentences into a numeric grade, a value of 1 is assigned to positive and improving sentences, and a value of 0 to negative sentences. After doing this, I computed a simple average of the points obtained in each subject in order to obtain a numeric grade ranging between 0 and 1. Multiplying by 10, this 0–1 grade was converted into a 0–10 grade, and it was translated afterwards into the five scale values with the same criteria outlined for secondary school grades. Based on this information, I constructed a summary measure of each student’s performance during the academic year. The main measure used throughout the analysis is the average grade obtained each academic year by each student. This measure is obtained by averaging the student’s grades in all subjects and terms in a given academic year.

## Appendix B

See Fig. 8 and Table 14.

**Table 14:** Effect of father’s job loss on the average grade: Alternative definitions

Dependent variable:	C.1	C.2	C.3	C.4	C.5	C.6	C.7
Average grade	FE	CS	VA	FE	CS	VA	FE-All
*Panel A: Loose definition of involuntary job loss*							
FJL	$-$ 0.127*	$-$ 0.218	$-$ 0.250***	$-$ 0.119*	$-$ 0.234	$-$ 0.229**	$-$ 0.092
	(0.071)	(0.139)	(0.082)	(0.062)	(0.149)	(0.108)	(0.058)
Average grade 2008			0.796***			0.943***	
			(0.041)			(0.066)	
Observations	890	178	178	780	178	123	1360
Students	178			178			332
Kinder excluded				$\checkmark$	$\checkmark$	$\checkmark$	
Student fixed effects	$\checkmark$			$\checkmark$			$\checkmark$
*Panel C: Job losses coming only from firm downsizes or firm/plant closures*							
FJL	$-$ 0.218***	$-$ 0.356**	$-$ 0.355***	$-$ 0.218***	$-$ 0.382**	$-$ 0.318***	$-$ 0.150**
	(0.074)	(0.166)	(0.094)	(0.068)	(0.178)	(0.103)	(0.073)
Average grade 2008			0.814***			1.013***	
			(0.043)			(0.068)	
Observations	770	154	154	671	154	105	1168
Students	154	154	154	154	154	105	285
Kinder excluded				$\checkmark$	$\checkmark$	$\checkmark$	
Student fixed effects	$\checkmark$			$\checkmark$			$\checkmark$

FJL (father’s job loss): dummy equal to 1 from the year the father loses the job. Average grade scaled to mean 0 with $SD = 1$ based on entire population of 358 students. *, **, *** denote significance at the 10%, 5% and 1% levels. Clustered robust standard errors at the family level in parentheses. There are 137 clusters (35 of them treated). All models include year and stage of education dummies. Except in Columns 1, 4 and 7, regressions include a dummy for father’s education, gender and the student’s quarter of birth. FE, fixed effects; CS, cross section of 2012; VA, value-added regressions

## Footnotes

1 Data computed from Labour Force Survey registers of total employment (annual averages) published by Eurostat.

2 See, among others, Jacobson et al. (1993), Kletzer (1998), Couch and Placzek (2010), Von Wachter et al. (2009), Huttunen et al. (2011), Barling et al. (1999), Eliason and Storrie (2009a), Eliason and Storrie (2009b), Eliason (2012), Charles and Stephens (2004) and Leombruni et al. (2013).

3 This school is an average school in Catalonia, both in terms of the academic performance of their students, and the socio-economic background of the parents. More details about this school are provided in Appendix A.

4 Unfortunately, the data do not include parental time use questions, to assess its potential importance as a mechanism driving the results.

5 See Björklund and Salvanes (2011) for a summary and evaluation of empirical research on education and family background.

6 I offer more details on the characteristics of the school as well as a comparison of the performance in standardised tests in this school with the Catalan average in Appendix A (Sect. A1.1).

7 In Appendix A, Sect. A.1.2, I provide more details on the characteristics of the sample compared to different population definitions in the Spanish Labour Force Survey.

8 Due to missing data for some individuals on the exact date of job loss, I had to make reasonable assumptions regarding the dates of job loss. Even if I was able to use the 5 years of information by making these assumptions, they could be introducing some measurement error in the dates regarding changes in employment. Robustness checks are performed using only the three points in time for which data are collected (2008, 2010 and 2012).

9 There is no scope to analyse whether job loss lead to separation in the sample, or to understand the impact of parental job loss in single parent households: only three mothers stated that they were single in 2008, and there are only eight cases in which the mother reports a separation or divorce after the 2008 academic year.

10 There is a majority of students that cannot be observed in 2008 because they were too young to be in school in the academic year 2007–2008. These are children that in the academic year 2012 were enrolled in the first grade of primary school or below. See Appendix A, Sect. A1.1 for a description of the Spanish education system. The remaining students that are not observed in academic year 2008 are those that entered the school during secondary school. That is, they graduated from primary school elsewhere and enrolled in the school where I collected the data once they moved to secondary school.

11 Note that in order to offer descriptive evidence for the maximum number of treated clusters, I use the loose definition of job loss in this section. The evidence is very similar using the strict definition of job loss.

12 This graph can be made more informative if the treatment group is split into students whose fathers lost their jobs in the first period (2009–2010) and students whose fathers lost their jobs in the second period (2011–2012). Figure 8 in Appendix B shows that compared to the control group, the group treated in the first period suffers a larger decrease in the average grade in the first period than in the second period. The group treated in the second period only experiences a significant decrease in the average grade with respect to the control group in the second period (once they have been treated with father’s job loss). These results would be offering suggestive evidence of a causal effect, but given the small sample size further splitting treated students in two groups is very demanding. Therefore, this evidence needs to be taken with caution.

13 The data for the home-ownership and household size dummies refer to 2012, and therefore, it could potentially be affected by treatment. Unfortunately, data for 2008 are not available. The data for 2012 are nonetheless reproduced at the end of Table 2 because it is discussed in Sect. 5.

14 Some of the variables in this table have missing information, so the number of observations available is shown in an additional (third) row. As a way to partially assess whether these missing observations are related to father’s job loss, I include a dummy in the table that is equal to 1 if income is missing. As it appears, there are no significant differences in the level of missing income information between treated and control students.

15 Note that the high-income category variable is derived from the following survey question: NET monthly income in euros (which includes salary, unemployment benefits, pension or other subsidies), with possible answers as follows: (1) less than 999 euros, (2) between 1000 and 1499 euros, (3) between 1500 and 1999 euros and (4) more than 2000 euros. The father is classified in the high-income category in 2008 if he marked options (3) or (4).

16 High motivation equals 1 if in 2008 the father had a level of motivation at work of 4 or 5 (measured in a Likert scale of 1 to 5, where 1 means ‘not motivated’ and 5 means very ‘motivated’).

17 Average grades are standardised using the mean and standard deviation of the whole sample. Standardisation is not performed at the year level, or at the year and cohort level, because the number of observations available for each group would be small. Instead, I use the average grade standardised based on the mean and standard deviation of all students in the sample, and control for year and stage of education dummies.

18 This assumption cannot be invoked when analysing the effect of getting back to employment after job loss. Nonetheless, Sect. 5.3 will explore whether the main results change when using alternative treatment definitions (i.e. allowing the treatment variable to switch back to 0 once the worker finds a new job).

19 However, it is important to note that the individual fixed effect controls for static pre-2008 labour market experience (i.e. number of years in unemployment prior to the Great Recession).

20 For instance, one might think that students from low socio-economic backgrounds would have experienced a different evolution of grades even in the absence of father’s job loss.

21 Ideally, one could include individual specific trends instead. However, given the sample size and the number of periods available, this option would be too demanding.

22
Ruiz-Valenzuela (2020) shows that parental job insecurity affects negatively the educational outcomes of their offspring.

23 Results in this section are very similar to those obtained when using the percentile rank of the students in their cohort as a dependent variable. The percentile rank is not used as the main measure for two reasons. First, I do not have data for the whole population in the school, which would be the ideal scenario. Second, the average grade has been used in other studies in the literature (Rege et al. 2011), and using it here will be useful to compare results.

24 See, for instance, Jacobson et al. (1993) or Farber (2011). These papers argue that these results are consistent with the destruction of job or industry specific human capital when a long-term job ends.

25 House not fully paid means that the household either lives in a rented house or has a mortgage. This information is only available for 2012, although ideally we would want this to be measured in 2008 values. However, it seems very unlikely that those families that are renting or paying a mortgage in 2012 would have had a fully paid house in 2008. Changes in housing status during the Great Recession in Spain are more likely to go from paying a mortgage to renting and the other way around. The way the housing variable is defined could be seen as a good proxy for housing status in 2008.

26 Specifically, ‘All FJL’ is a dummy variable that equals 1 the year the father loses the job and the years after job loss as long as the father remains unemployed, and it equals 0 when the father is employed. ‘FJL leading to long-term unemployment’ is a dummy variable that equals 1 the year the father loses the job (as long as he does not find a job the same year), and the years after job loss as long as the father remains unemployed. It equals 0 when the father is employed. The only difference between ‘All FJL’ and ‘FJL leading to long-term unemployment’ is that ‘All FJL’ considers fathers during the year of job loss as treated, whereas ‘FJL leading to long-term unemployment’ only considers them as treated as long as they do not find a job during the same year.

27 The loose definition of job loss is used here to maximise the number of treated clusters, given that I am further splitting the treated group in two subgroups in each regression. Results are very similar when using the strict definition of job loss.

28 People with low levels of education are less likely to find a job, as could be seen by using data from the Spanish Labour Force survey. Even if according to these data low-educated fathers have a higher probability of suffering long unemployment spells after job loss, the results in Sect. 5.3 are not stemming from the same group of students. The correlation between a dummy variable for low-educated fathers and a dummy variable indicating long-term unemployment is not significant and close to zero.

29 Even if not shown, the impact of FJL seems to be concentrated on poorer households, as given by father’s net income in 2008. However, standard errors are very large in this case.

30 Ideally, all the variables interacted with FJL should be measured in 2008 values. For certain variables, the data were not available and the 2012 has been used as an approximation of the 2008 value in Columns 5 and 6.

31 However, it is important to note some important differences between both papers. First, students in Rege et al. (2011) are generally older (16 years old). Second, the estimation strategies used by the two papers differ. While Rege et al. (2011) use a pooled cross section of 10th graders and job losses coming from plant closures, this paper uses within-individual variation to analyse job losses that are mostly due to the Great Recession in Spain. Moreover, I cannot rule out the possibility that the larger magnitude of the results is due to particularities of the Spanish economy (different welfare and protection systems after job loss, for instance).
